# Radiofrequency Ablation (RFA) for Osteoid Osteoma in an 11-Year-Old Male Child With Sickle Cell Trait: A Case Report

**DOI:** 10.7759/cureus.63626

**Published:** 2024-07-01

**Authors:** Nimmanagoti Nagaraju, Ashish Varma, Amar Taksande, Harshitha Reddy, Chaitanya Kumar Javvaji, Manasa Suryadevara, Naramreddy Sudheesh Reddy

**Affiliations:** 1 Pediatrics, Jawaharlal Nehru Medical College, Datta Meghe Institute of Higher Education and Research, Wardha, IND; 2 Internal Medicine, Jawaharlal Nehru Medical College, Datta Meghe Institute of Higher Education and Research,, Wardha, IND; 3 Radiodiagnosis, Jawaharlal Nehru Medical College, Datta Meghe Institute of Higher Education and Research,, Wardha, IND; 4 Pediatrics, Jawaharlal Nehru Medical College, Datta Meghe Institute of Higher Education and Research,, Wardha, IND

**Keywords:** prostaglandin, nonsteroidal anti-inflammatory drugs (nsaids), osteoid osteoma, sickle cell trait, radiofrequency ablation

## Abstract

Osteoid osteoma is a benign bone tumor that typically presents with nocturnal pain alleviated by nonsteroidal anti-inflammatory medications. The coexistence of osteoid osteoma with sickle cell anemia, a hereditary hemoglobinopathy characterized by vaso-occlusive crises and bone infarcts, poses diagnostic and therapeutic challenges due to overlapping clinical and radiological features. This condition primarily involves the long bones of the lower extremities, particularly the femur and tibia. Despite its benign nature, osteoid osteoma can significantly impact a patient's quality of life due to persistent and intense pain, often leading to substantial sleep disturbances and functional limitations.

## Introduction

A single aberrant haemoglobin beta gene (HbAS) allele characterizes sickle cell trait (SCT), a genetic condition typically considered benign and asymptomatic. However, SCT can occasionally lead to complications under certain physiological conditions, such as intense physical exertion, hypoxia, or dehydration [1]. Osteoid osteomas, first described by Jaffe in 1935, are relatively common, accounting for 12% of benign bone tumours. These tumours predominantly occur in the cortex of the diaphysis of the tibia or femur in over half of the cases. Osteoid osteomas are benign yet painful bone tumours that usually affect young individuals. The hallmark of this condition is a small, radiolucent nidus surrounded by sclerotic bone, primarily found in the long bones of the lower limbs [2].

Osteoid osteomas are more prevalent in males than females and can develop in the mature skeleton up to the age of 70, although 75% of cases occur between the ages of 5 and 25. The characteristic symptom of osteoid osteoma is nocturnal pain, which is significantly alleviated by nonsteroidal anti-inflammatory drugs (NSAIDs). Excessive prostaglandin production from the lesion leads to localized inflammation and vasodilation, exacerbating the pain. NSAIDs reduce this prostaglandin burden by blocking the arachidonic acid pathway, thereby decreasing the pain. Occasionally, osteoid osteomas induce hyperemia, resulting in abnormal bone growth and synovitis when associated with the joint and its capsule [3].

The co-occurrence of SCT and osteoid osteoma is rare, presenting unique diagnostic and therapeutic challenges. This case report aims to detail a patient with SCT diagnosed with osteoid osteoma, highlighting the clinical presentation, diagnostic process, and management strategies employed. The objective is to provide insight into the potential challenges and considerations when treating patients with SCT and bone tumours. The etiology of osteoid osteoma remains unknown, but one hypothesis suggests it may arise from localized bone development abnormalities or inflammation. Histologically, the lesion comprises a core nidus of osteoid and woven bone surrounded by a sclerotic reaction of the cortical bone. Diagnostic imaging, particularly computed tomography (CT) scans, is crucial for identifying the nidus and distinguishing osteoid osteoma from other osteoblastic conditions.

## Case presentation

**Figure 1:**
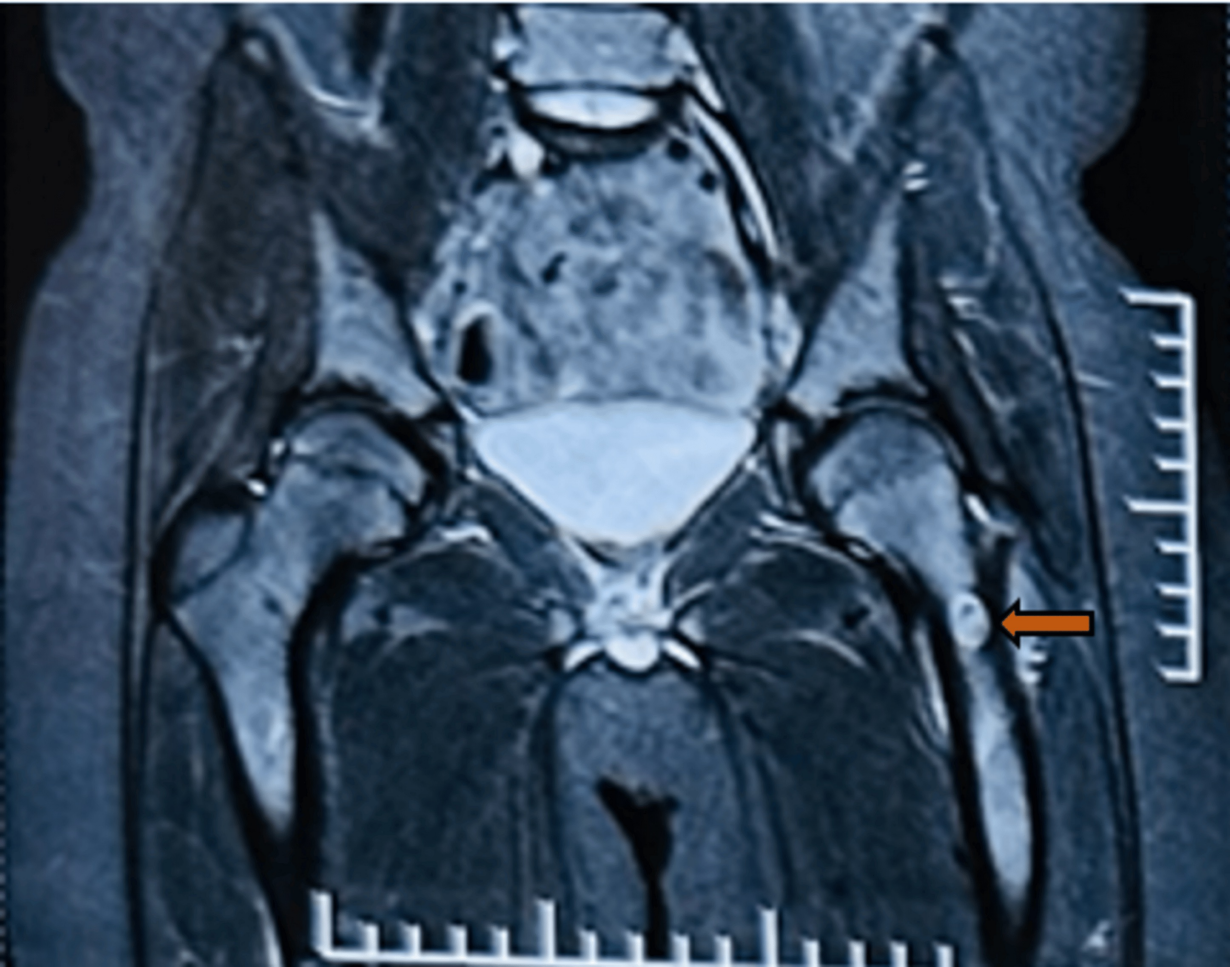
Magnetic resonance imaging of the hip, coronal section showing oval hyperintense lesion in the neck of left femur suggesting the possibility of osteoid osteoma (Orange arrow)

An 11-year-old male came with complaints of left leg pain for one year. According to his mother, he had been apparently in normal condition until a year back, then had complaints of sudden onset of sharp and intense pain in the left upper thigh, severe in grade, non-radiating, which was notably worse in the morning. The pain was not associated with fever and was alleviated by high doses of analgesics. Despite consultations with various local practitioners, the symptoms persisted. Consequently, the parents sought advice from an orthopaedician, who recommended a magnetic resonance imaging (MRI) of the hip. The MRI coronal section of the hip revealed an oval hyperintense lesion in the neck of the left femur, suggesting the presence of an osteoid osteoma (Figure 1).

The child was referred to our tertiary care centre for further management. The child was diagnosed with SCT at the age of 1 year. The child has no history of blood transfusions or hospitalizations. Upon physical examination, pallor and inguinal lymphadenopathy were noted, with no signs of clubbing, icterus, or cyanosis. Vital signs included a pulse rate of 102 bpm, a respiratory rate of 22 bpm, a blood pressure of 98/68 mmHg, and an oxygen saturation of 98% on room air. An abdominal examination revealed splenomegaly and hepatomegaly, with no other significant findings. Other systems examinations were normal. Laboratory investigations (complete blood count, liver function tests, kidney function tests, and coagulation profile) were within normal limits.

**Figure 2:**
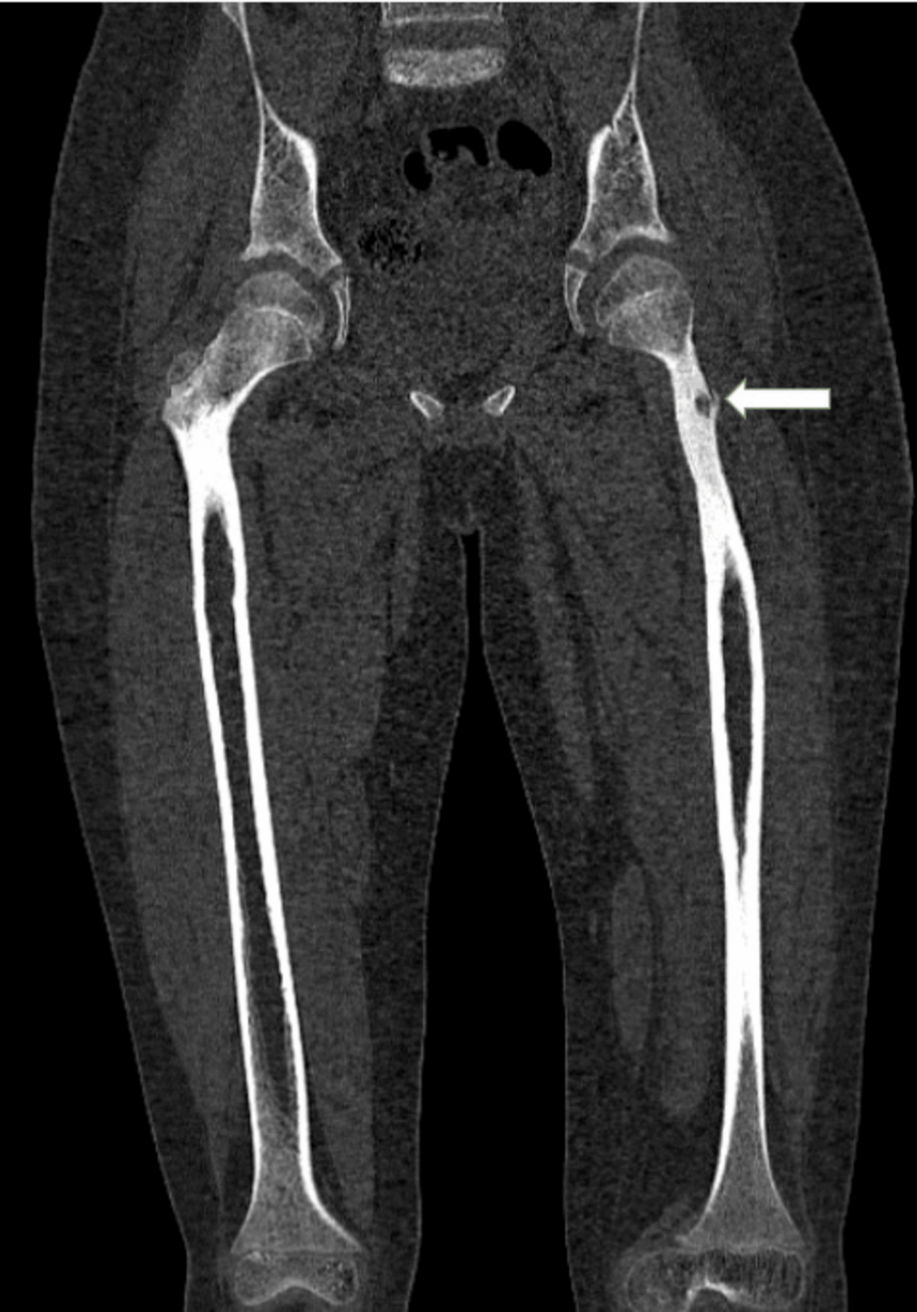
CT post radio frequency ablation revealing evidence of a well-defined lytic lesion with adjacent cortical sclerosis and anterior cortical break in the neck of the left femur showing punctate calcific focus within (white arrow)

The patient was referred to an interventional radiologist who recommended radiofrequency ablation (RFA) for the osteoid osteoma. Under aseptic conditions, an 11G bone biopsy needle was used to access the lesion in the left femoral neck. An RFA probe was positioned within the lesion through the biopsy needle. After confirming the position of the probe tip within the lesion, RFA energy was delivered at 30 watts, targeting a temperature of 90°C for 2 minutes. The procedure was uneventful. CT post radio frequency ablation revealed a well-defined lytic lesion with adjacent cortical sclerosis and an anterior cortical break in the neck of the left femur, measuring approximately 14 x 8 mm, with a punctate calcific focus within (Figure 2). The patient remained vitally and hemodynamically stable and was subsequently discharged with advice for follow-up.

## Discussion

Osteoid osteoma is a benign but painful bone tumour that commonly affects young individuals, predominantly males. Characterized by a small size, typically less than 2 cm in diameter, it often manifests with nocturnal pain that responds well to NSAIDs. While its pathophysiology and clinical presentation are well-documented, the co-occurrence of osteoid osteoma with other conditions can present unique challenges in diagnosis and management [2]. Sickle cell anaemia, a hereditary hemoglobinopathy, is one such condition that can complicate the clinical picture. Patients with sickle cell anaemia often present with bone pain and vascular complications, which can obscure the symptoms of coexisting musculoskeletal conditions. The diagnostic difficulty is exacerbated by overlapping clinical symptoms and radiologic findings, requiring a high level of suspicion and a thorough diagnostic assessment [4].

There are various reports of malignant transformation. Many people who are affected have experienced excruciating lesion-related pain, which reduces in intensity at night and is reduced in intensity with NSAIDs [3]. RFA has become a popular and successful minimally invasive therapy option for osteoid osteoma. By inducing localized tissue necrosis through thermal energy, this treatment offers quick pain relief and functional recovery [5]. However, applying it to sickle cell anaemia patients necessitates carefully weighing the procedure's possible dangers against the patient's overall hematologic state.

This case study details the effective use of RFA to treat an osteoid osteoma in a sickle cell anaemia patient. It draws attention to the subtleties of the diagnostic process, the procedural strategy, and the clinical results, offering insights into the interactions between these two circumstances and the effectiveness of RFA in this particular clinical setting. This method allows for accurate heat distribution to the targeted tissue under the guidance of pictures. A delivery probe transmitting a high-frequency alternating current at 500,000 Hz causes localized ionic agitation and frictional heat in the surrounding tissue, which eventually results in coagulation necrosis. The threshold for osteocyte necrosis in animal models is 50°C administered for 30 seconds, with blood flow-induced cooling limiting the necrosis zone [6].

Technological innovations in ablative therapy complemented by modern surgical techniques have revolutionized the treatment of osteomas. With simple, low-cost equipment, CT-guided drilling allows minimally invasive methods. Case studies indicate that adding ethanol instillation into the nidus is promising; nonetheless, more research is necessary. Compared to radiofrequency therapy, interstitial laser therapy with minimum access enables regulated coagulative necrosis in an outpatient population with a tolerable but higher rate of mild complications [7].

## Conclusions

This case study demonstrates the successful use of RFA to treat an osteoid osteoma in an 11-year-old patient with SCT. The co-occurrence of these conditions posed significant diagnostic challenges due to overlapping symptoms such as bone pain and radiologic findings. However, by means of a thorough diagnostic process, including clinical evaluation, imaging, and laboratory investigations, the osteoid osteoma was accurately identified. RFA a minimally invasive procedure, provided effective pain relief and rapid functional recovery by precisely targeting and inducing localized tissue necrosis within the tumor. This case underscores the importance of tailored treatment approaches in managing complex conditions and supports the efficacy of RFA as a viable option for patients with benign bone tumors complicated by SCT.
